# Biofabrication methods for reconstructing extracellular matrix mimetics

**DOI:** 10.1016/j.bioactmat.2023.08.018

**Published:** 2023-09-09

**Authors:** Abdellah Aazmi, Duo Zhang, Corrado Mazzaglia, Mengfei Yu, Zhen Wang, Huayong Yang, Yan Yan Shery Huang, Liang Ma

**Affiliations:** a State Key Laboratory of Fluid Power and Mechatronic Systems, Zhejiang University, Hangzhou, 310058, China; b School of Mechanical Engineering, Zhejiang University, Hangzhou, 310058, China; c Department of Engineering, University of Cambridge, Cambridge, United Kingdom; d School of Medicine, The Chinese University of Hong Kong, Shenzhen, Guangdong, 51817, China; e The Affiliated Stomatologic Hospital, School of Medicine, Zhejiang University, Hangzhou, 310003, China; f Center for Laboratory Medicine, Allergy Center, Department of Transfusion Medicine, Zhejiang Provincial People's Hospital, Affiliated People's Hospital, Hangzhou Medical College, Hangzhou, Zhejiang, 310014, China

**Keywords:** Extracellular matrix, Biofabrication, Electrospinning, Bioprinting, Organ-on-a-Chip

## Abstract

In the human body, almost all cells interact with extracellular matrices (ECMs), which have tissue and organ-specific compositions and architectures. These ECMs not only function as cellular scaffolds, providing structural support, but also play a crucial role in dynamically regulating various cellular functions. This comprehensive review delves into the examination of biofabrication strategies used to develop bioactive materials that accurately mimic one or more biophysical and biochemical properties of ECMs. We discuss the potential integration of these ECM-mimics into a range of physiological and pathological *in vitro* models, enhancing our understanding of cellular behavior and tissue organization. Lastly, we propose future research directions for ECM-mimics in the context of tissue engineering and organ-on-a-chip applications, offering potential advancements in therapeutic approaches and improved patient outcomes.

## Highlights

• ECM are dynamic structures undergo continuous changes.
• ECM can be mimicked using natural polymers, synthetic polymers or decellularized ECM.
• Four biofabrication methods can be used to construct the ECM-mimics.
• Distinct ECM-like models have been developed in line with the specific function and structure of the original tissue.

## Graphical abstract

**Figure:**
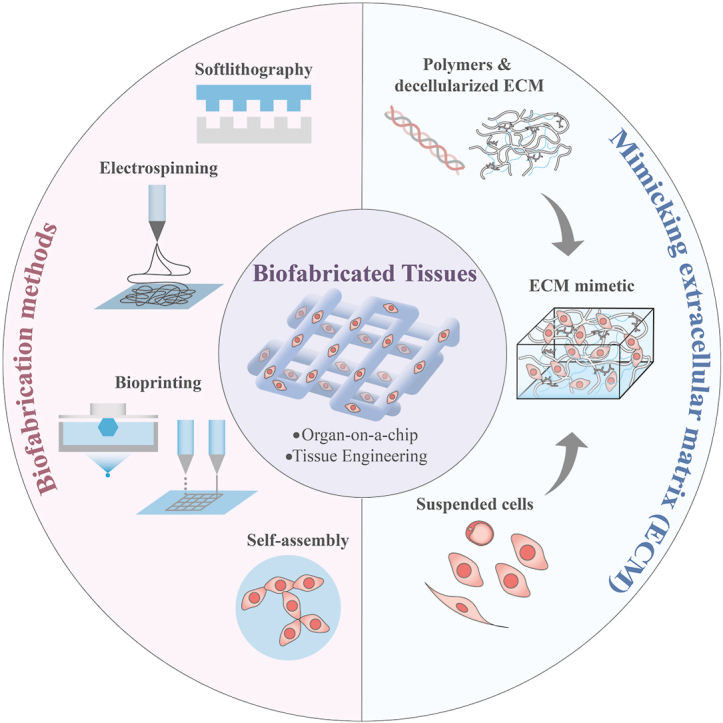

## 1 Introduction

The recapitulation of cellular habitats at numerous length scales is widely regarded as the key to fabricating functional, complex tissue models in the laboratory, as cells' interactions with the external environment determine their behaviors, including migration, proliferation, and apoptosis. Such interactions are achieved through binding specific transmembrane receptors and extracellular recognition motifs, which in turn triggers physiological responses through signal transduction [1,2]. Significantly, cells perceive and react not only to their microenvironment's stiffness but also to the spatial configuration of chemical and physical cues. These cues might be uniformly distributed, grouped, or patterned and can be laid out on a wide range of substrates – flat two-dimensional surfaces, textured surfaces, or three-dimensional matrices [[3], [4], [5]]. Thus, it has long been recognized that materials and tissue models with appropriate physical and biochemical cues from the extracellular matrix (ECM) are essential in tailoring cell-matrix and cell-cell interactions [[6], [7], [8]].

The originally used model for *in vitro* study is the monolayer of cells in petri-dish cell culture. Although the two-dimensional (2D) cell culture model has made significant contributions to biological research, it has been proved to have poor representations of the cellular interactions that take place *in vivo* [9]. Animal models have been an alternative option. However, the use of animal models and humans in research has often been confined by the availability of test subjects, the feasibility of testing procedures, and ethical concerns about discomfort or pain caused to live subjects. In addition, animal models might not precisely predict the clinical efficacy of therapeutics for certain human tissue types [9]. To mitigate the constraints associated with the use of animal models, the disciplines of tissue engineering and organs-on-chips have pivoted towards the development of materials and structures that emulate the ECM and the refinement of biofabrication techniques. The objective is to construct highly accurate *in vitro* 3D tissue models, providing a basis for methodical, reproducible, and quantifiable investigations. In parallel, organs-on-chips technologies strive to emulate dynamic physiological environments, adding an additional layer of complexity and realism to the tissue models [10,11]. These developments promise to greatly contribute to the clinical translation of tissue engineering research and potentially revolutionize the drug development pipeline.

In this review, we systematically introduce extracellular matrix and its correlation with different biomaterials and various types of cutting-edge biofabrication strategies in recapitulating ECM features, including soft lithography, electrospinning and bioprinting. This review offers a full-scale knowledge hierarchy in developing biofabrication strategies from simple cell culture substrates towards complex tissue and organ models. In addition, this review discusses the latest applications in fabricating ECM-mimic tissue and organ models in multiple length scales, including the brain, blood-brain barrier, heart, liver, bone, and cancer models. Finally, this review emphasizes the potential of complex ECM-mimicking tissue and organ models and provides a guideline for applying biofabrication strategies to advancing biomedical research and clinical applications.

## 2 Extracellular matrices and their functions

**Fig. 1:**
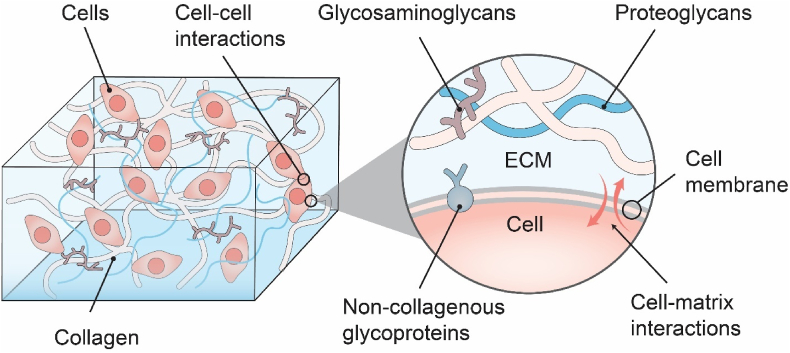
Schematics of the Extracellular Matrix (ECM) illustrating the structural and chemical complexity. Different components within ECMs link together to form mechanically stable composites, and correlate to cellular metabolisms and migrations.

Extracellular matrices (ECMs) are non-cellular complex polymer networks found naturally in animal tissues that are secreted by cells [12]. Their main components are collagen, non-collagenous glycoproteins, proteoglycans, and glycosaminoglycans (Fig. 1). Moreover, ECMs are highly dynamic structures that undergo continuous enzymatic and non-enzymatic remodeling, and their molecular constituents go through a myriad of post-translational changes [12,13]. Based on the structural and chemical complexity, ECMs could be seen to structurally exist with a phase of fibril architecture and a phase of hydrated interstitial gel [14].

The extracellular matrix reflects the mechanical properties of each type of tissue and organ, such as elasticity, strength, and stiffness; they are protected by a buffering action that maintains extracellular homeostasis and water retention [13,15,16]. In addition, ECMs provide essential physical scaffolding for the cellular constituents; different components within ECMs usually link together to form structurally stable composites, contributing to the mechanical properties of tissues [8,16,17]. The following sessions will explain major ECM functions in detail.

### 2.1 ECM remodeling and storing functions

ECMs are constantly undergoing remodeling, through which ECMs’ components are deposited, degraded, or modified. Intermolecular cross-linking by Lysyl Oxidases (LOX) is a key posttranslational modification for collagens and elastin. Expanded cross-linking due to excess LOX activity increases tissue tensile strength and matrix stiffness and, in turn, affects cellular behaviors [18]. Collagens and other ECM elements are substrates for A Disintegrin and Metalloproteases (ADAMs), Matrix Metalloproteinases (MMPs), as well as proteases such as cathepsin G and elastase [17,19]. MMPs are produced in precursor forms and remain inactive until activated. Most of the MMP family members are secreted in ECMs. Their activities are counteracted by tissue inhibitors of MMPs (TIMPs) and other inhibitors, and an imbalance may lead to tissue fibrosis and diseases [17,19].

Besides ECM molecules, MMPs and others can also cleave precursor proteins, release ECM-bound growth factors, and release from ECM proteins bioactive fragments with new bioactivities, such as endostatin [20]. ECMs are also influenced by biological stimuli, such as cytokines and glucocorticoids, and physical stimuli, such as oxidative stress, pressure, and mechanical stretch. Among these, the most studied cytokine is transforming growth factor-β (TGF-β), which enhances ECM production and upregulates ECM-related genes. In addition to remodeling, ECMs usually act as reservoirs to store and sequester cytokines and growth factors. In particular, the fibroblast growth factor family strongly binds to heparan sulfate chains of proteoglycans; heparan sulfate proteoglycans are also involved in binding, transporting, and activating developmental control factors, including wingless-related integration site (Wnt) factors and hedgehog [21]. ECMs are reservoirs of bioactive fragments released upon limited proteolysis; these fragments regulate physiological and pathological processes, including angiogenesis. ECMs participate in ligand maturation; TGF-β, which is secreted in latent form, is stored in the ECM and remains inactive until activated by MMP-dependent proteolysis [19].

### 2.2 ECM correlates to cellular behaviors and cell migration

ECMs affect some of the most fundamental behaviors and characteristics of cells, such as proliferation, adhesion, migration, polarity, differentiation, and apoptosis [18,19]. The biochemical properties of ECMs allow cells to sense and interact with their extracellular environment through various signal transduction pathways. Such biochemical cues are provided by ECM components, including adhesive proteins such as fibronectin, integrin/non-integrin receptors, growth factors, and associated signaling molecules. Interactions with different matrices via specific sets of receptors can trigger distinct cellular responses [22,23].

Besides biochemical cues, ECMs also play essential roles as physical barriers, anchorage sites, or movement tracks for cell migration [19]. The physical properties of an ECM, including its rigidity, density, porosity, insolubility, and topography (spatial arrangement and orientation), provide physical cues to the cells. The mechanical properties are essentially sensed by integrins that connect extracellular ECM to the actin cytoskeleton inside the cells. Stiff matrices induce integrin clustering, robust focal adhesions, Ras homologous (Rho) and mitogen-activated protein (MAP) kinase activation, leading to increased proliferation and contractility. Matrix rigidity also regulates differentiation. For example, mesenchymal stem cells favor a neurogenic path on soft matrices, and on stiff ones, they favor an osteogenic path [21].

## 3 Chemically-defined ECM and synthetic ECM analogues

Both natural and synthetic polymeric materials are commonly used in tissue engineering due to their low cytotoxicity and structural similarity to the extracellular matrix (ECM) [24,25]. The highly hydrated network structure permits the exchange of gases and nutrients and makes them an attractive option for the formation of ECM mimics. Scientists have summarized the fundamental structural and chemical features of ECM and focused on the materials side, especially the multiple chemical synthetic strategies in bioconjugation and polymer crosslinking, in which ECM-mimic bioresponsive polymers are produced for cell cultures and tissue engineering applications [26]. As illustraed in Table 1, essential qualities of ECM-mimicking materials include biocompatibility, mechanical integrity, biodegradability, and multi-functionality. In addition, integrating such materials provides opportunities for combining components to tailor the overall tissue engineering materials toward facilitating specific requirements [27,28].

**Table 1:** Examples of different types of materials used to mimic ECM.

Biomaterials	Method of fabrication	Cell types	Specifications
**PEG**	Soft lithography	CMs	Alignment of the focal adhesions [258]
**PS**	Soft lithography	Human AM-MSCs + mouse ESCs	Early differentiation of mESCs and heterogeneous cells [259]
**PHB**	Electrospinning	MSCs, CMs, CFs	Induced angiogenesis, reparative process and remodeling [260]
**BSA/PVA**	Electrospinning	Human MSCs	Cardiogenic differentiation of MSCs [261]
**PMGI + heparin-binding peptide I**	Electrospinning	HeLa, human PSCs	Enhanced HeLa cell attachment and potentiated CM differentiation of hPSCs [262]
**PGS/gelatin**	Electrospinning	CMs	Superior mechanical properties, enhanced CM beating properties [263]
**PLGA + YIGSR**	Electrospinning	Neonatal rat CMs	Higher expression of a myosin and b-tubulin, faster and latest longer contraction of CMs [264]
**PCL + azacytidine**	Electrospinning	Human MSCs	*In vitro* cardiac differentiation of hMSCs [265]
**Cellulose + CS/SF**	Electrospinning	AD-MSCs	Reduced ventricular remodeling post-MI [266]
**PCL:PGA**	Electrospinning	CPCs	Cell attachment and differentiation *in vitro* and support living cells *in vivo* [267]
**Porcine spinal cord-derived ECM** **PCL**	Electrospinning	human neuroblastoma cell line (SH-SY5Y)	ECM fiber scaffolds promote the migration of mature neurons after lesion; provide biochemical and topographical cues to guide the migration of mature neurons [268]
**Fibronectin**	Electrospinning	Bone murine stromal cells ST-2 cell line	Functionalizing PCL electrospun mats with fibronectin (surface entrapment method), resulting in the best cell response [2]
**PCL**	Electrospinning	Human osteoblast-like cells (MG-63)	Variation in surface characteristics leading to increased cell adhesion and collagen mineralization on porous fibers; Negative zeta potential of PCL sample promoted calcium mineralization crucial for tissue formation [269]
**PEG-GelMA**	Inkjet bioprinting	Bone marrow-derived human MSCs	Enhanced osteogenic and chondrogenic differentiation; improved gene and protein expression analysis [270]
**Collagen**	Extrusion bioprinting	Human corneal epithelial cell line (HCE-T)	Cornea-like structure with keratocytes demonstrating high cell compatibility [271]
**Hyaluronic acid**	Extrusion bioprinting	Human glial cell	Brain microenvironment and Glioblastoma invasion [272]
**Alginate- gelatin**	Extrusion bioprinting	Animal fibroblast cells	Unique structures with varied naproxen coating, with increased tensile strength and biocompatibility [273]
**GelMA**	Extrusion bioprinting	Human ADSCs	GelMa substrates with Pore size and foamability are controlled by processing parameters [274]
**HAMA**	Extrusion bioprinting	Human bone marrow-derived MSCs	Increase in mechanical stiffness and long-term stability; Useful in creating porous and anatomically shaped scaffolds [275]
**PCL**	Extrusion bioprinting	Human bone marrow MSCs	Personalized and implantable hybrid active scaffolds for critical-size bone defects; Zigzag/spiral PCL cage proved to be mechanically strong with sufficient nutrient/gas diffusion [276]
**Porcine skin-derived ECM Nano-hydroxyapatite Gelatin Quaterinized chitosan**	Extrusion bioprinting	ADSCs, human bone marrow-derived MSCs and HUVECs	Antibacterial, hemocompatible, and biocompatible; Promoted cell attachment and proliferation, osteogenesis and vascularity regeneration [277]
**Ovine aortic valve derived ECM Gelatin Alginate**	Extrusion bioprinting	Ovine valvular interstitial cells	dECM hydrogel impaired HUVEC viability [278]
**PEG8NB**	SLA(DLP) bioprinting	Pancreatic cancer cells (COLO-357), NIH 3T3 fibroblasts, and mouse MSCs	High precision and cell compatibility. Enable the creation of diverse bioprinted constructs [279]
**Dental follicle-derived ECM** **GelMA**	SLA (DLP) bioprinting Extrusion bioprinting	Human dental follicle cells	GelMA/dECM module promotes periodontal tissue regeneration; enhancement in bone–ligament interface fusion, and periodontal fiber alignment [280]
**PA/PBS** **PA/CaCl2 solution**	Self-assembly driven (shear）	Bone marrow-derived human mesenchymal stem cells	Ability to bundle and align microfibres and tube formation by constraint Assembly into microfibres [281]
**Polymer-based hydrogels**	Self-assembly driven (magnetic）	NIH 3T3 Mouse fibroblasts cell line	Cell-friendly and touch-free organization of microgels Compatibility with a range of materials + uses magnetism of cells directly [282]
**Polymeric solution**	Self-assembly driven (Liquid–Liquid attraction/Immiscibility)	NIH 3T3 Mouse fibroblasts cell line	Cell-friendly and touch-free organization of microgels Complex shapes through delicate interactions [283]
**PA/ELP**	Self-assembly driven (supramolecular）	Primary mouse- ADSCs and HUVEC	Self-driven assembly into a tubular shape Selective presentation and density of epitopes [284]

*Abbreviations:* PEG- *Poly(ethylene glycol)*; CM-*cardiomyocytes*; PS- *Polystyrene*; AM-MSC- *Amniotic membrane-derived mesenchymal stem cells*; ESC- *embryonic stem cells*; PHB-*Poly(3-hydroxybutyrate)*; CF- *Cardiac fibroblasts*; BSA/PVA- *Bovine Serum Albumin/Poly(vinyl alcohol)*; PMGI- *Polymethylglutarimide*; hPSC- *human Pluripotent stem cells*; PGS- *Poly (Glycerol Sebacate)*; PLGA*-Poly(lactic-co-glycolic acid)*; YIGSR- *Tyr–Ile–Gly–Ser–Arg*; PCL- *Poly(ε-caprolactone)*; MSC*- mesenchymal stem cells*; CS/SF- Chitosan/silk fibroin; AD-*Adipose tissue*; PGA-Poly(glycolic acid); CPC-*Cardiac progenitor cells*; GelMA- *Gelatin-Methacryloyl*; HAMA- *Hyaluronic acid methacrylate*; ASC-*Adipose stem cells*; HUVEC- *human umbilical endothelial cells*; SLA-*Stereolithography*; DLP-*Digital light processing*; ECM-*Extracellular matrix*; dECM-*decellularized extracellular matrix*; PA-*peptide amphiphiles*; PBS- *Phosphate-buffered saline*; ADSC- *primary mouse-adipose-derived stem cells. ;* ELP- *Elastin-like polypeptide*.

### 3.1 Natural polymers mimicking ECM

Natural polymers, also called bio-derived materials, occur naturally and can be extracted using physical or chemical methods [29]. Naturally occurring polymers include silk, wool, deoxyribonucleic acid (DNA), cellulose, and proteins. Some natural polymers, such as gelatin, alginate, fibrinogen, and hyaluronic acid (or hyaluronan), are water-soluble, which implies they can dissolve in cell-friendly inorganic solvents, such as cell culture medium and phosphate-buffered saline, to form solutions/hydrogels. The solution or hydrogel states of the natural polymers hold certain fluidity enabling them to be 3D printed layer-by-layer under the instructions of (Computer Aided Design) CAD models [[30], [31], [32]]. The polymeric solutions/hydrogels offer cells and biomolecules (i.e., bioactive agents) a mild environment, similar in composition to the ECM, facilitating cellular activities (i.e., cell-response bioactivities). However, polymers like polysaccharides and proteins need functionalization, which can be achieved by attaching bound functional groups to create structurally conformed ECM structures. Thiol-Michael and Diels-Alder additions are the most used reactions to functionalize these natural biomaterials through “click” chemistry.

Only a few natural polymers can be printed in layers at benign cell conditions (such as room temperature) without the help of physical-chemical crosslinking of the incorporated polymer chains. Thus, very few natural polymers can meet all the basic requirements for tissue/organ scaffold bioprinting [[33], [34], [35]]. During and after the bioprinting process, natural polymers have played several essential roles in multiple cellular/biomolecular interactions, homogeneous/heterogeneous histogenesis modulations/integrations/coordinations, and bioartificial organ generations/maturations. These essential roles include providing suitable accommodation for cellular and biomolecular activities (e.g., growth, migration, aggregation, proliferation, differentiation/mobilization, infiltration, coaction), enough space for extracellular matrix (ECM) patterns (e.g., formation, secretion, orientation), biophysical/chemical cues for tissue/organ morphologies (e.g., formation, modeling, reshaping), and hierarchical vascular/neural/lymphatic network settings (e.g., construction, integration, figuration) [29].

### 3.2 Synthetic polymers in reconstructing ECM features

Besides natural polymers, synthetic polymers are also widely used in biofabrication, including polyethylene glycol (PEG) [[36], [37], [38], [39]], polycaprolactone (PCL) [[40], [41], [42]], polyvinylpyrrolidone (PVP) [[43], [44], [45]], poly(l-lactic) acid (PLA) [[46], [47], [48]] and poly(lactic-co-glycolic) acid (PLGA) [49]. They can be tuned to comply with tissue-specific degradation and mechanical property requirement of the target tissues and organs. Although some of the limitations (use of toxic solvents, melting points higher than body temperature, difficulty in encapsulating cells) might hinder their translational applications, synthetic polymers have still been developed as biological substitutes to address many drawbacks of using natural biopolymers in potential therapeutic applications [50]. For example, obtaining regulatory approval for new biological therapies and medical devices based on cellularly secreted ECM components is challenging due to quality control and heterogeneity within a single cell population [51,52]. Additionally, problems associated with using the same suppliers or lot-sourcing with batch-variable natural biomaterials impede the replication and scalability of promising results. For instance, the polymerization, mechanical, and transport properties of bovine, porcine, and murine type I collagen are markedly different. Furthermore, two batches of porcine gelatin may even vary in their isoelectric point [53]. While potentially requiring more extensive synthesis or purification equipment, the workup of synthetic polymers that start from purified and uniform chemical reagents largely avoids the aforementioned sourcing problems.

Synthetic polymers have the advantage of tunable biophysical properties, which can investigate how cells react to each of these properties. As a result of their tunable properties, they can provide a broader range of tissue construction applications. However, they are typically inert to cell adhesion. They must be extensively functionalized with adhesive peptides or protein fragments, such as Arginyl-glycyl-aspartic acid (RGD) (fibronectin domain-mimicking) peptides and Ile-Lys-Val-Ala-Val (IKVAV) (laminin domain-mimicking) peptides, or used in tandem with biologically derived growth factors to achieve sufficient interaction with membrane-bound cellular proteins and allow for healthy metabolism of the cell population. Moreover, synthetic polymers usually lack sites for cellular recognition and other biological cues found in natural ECM for promoting cellular proliferation and differentiation. Though the functionalization of synthetic polymers can improve their biological properties, the presence of adaptable side groups becomes a prerequisite for proper customization of a construct's mechanical and biological properties [54].

### 3.3 Decellularized ECM

Decellularized ECM (dECM) is one of the closest replicates of the native ECM compositions. It is derived from native tissues, which are processed to eliminate materials associated with the inhabiting cells while maximally retaining the insoluble ECM components [55]. Since the main insoluble components of ECM, such as collagen, fibronectin and laminin, are largely conserved across multiple species, dECM scaffolds fabricated from readily available xenogeneic ECM sources offer potentially compatible biological signals across multiple species. They may act as an appropriate *in vitro* scaffold niche for maintaining human cell functions [56]. In addition, recent investigations in stem cell niches have suggested the importance of developing an *in vitro* stem cell microenvironment for cell expansion and tissue-specific differentiation. Scientists have discovered that decellularized stem cell matrix (DSCM) in stem cell niches may provide an expansion system to yield large-quantity and high-quality cells for cartilage tissue engineering and regeneration [57].

Whilst whole organ decellularization shows tremendous potential for tissue engineering and xenogeneic transplantation, dECM materials used for *in vitro* culture models are generally reduced to the forms of coating, hydrogels, or fiber mats of low dECM contents [58]. The inability to tailor the fibrous matrix's biochemistry, topography, ultra-structures, and poor availability presents a technology gap for fabricating more complex tissue models *in vitro*. Pati and his colleagues, on the other hand, used a combination of dECM and PCL, PLGA, and tricalcium phosphate (TCP), which gave the dECM tunable properties [59]. As a result, combining different types of biomaterials opens up new opportunities for providing better environments and structures for biofabricated tissue models.

## 4 Biofabrication methods to recapitulate ECM features and architectures

**Fig. 2:**
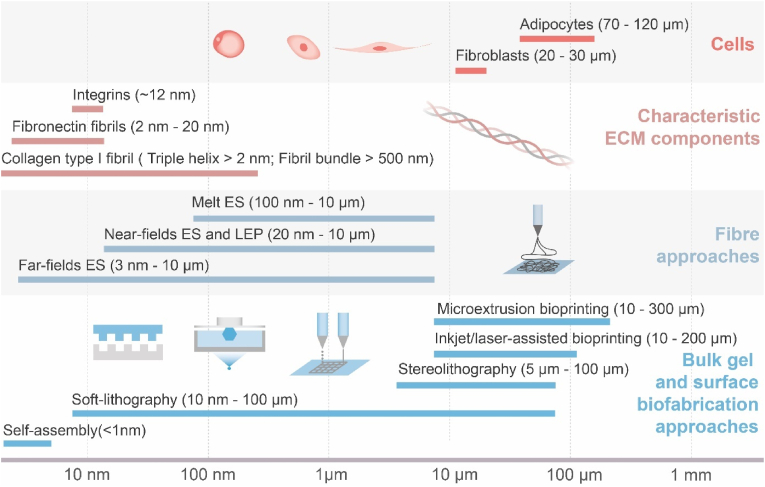
A length scale bar illustrating featured resolutions of various state-of-the-art biofabrication techniques in comparison with geometric sizes of representative cells and tissues [14,[63], [64], [65], [66], [67], [68], [69], [70], [71], [72], [73], [74]]. Herein ‘ES’ stands for electrospinning, ‘LEP’ stands for low-voltage electrospinning patterning.

Biofabrication is a multidisciplinary field that integrates knowledge from medicine, biology, and materials science to develop artificial hydrogel-based *in vitro* organ models and alternatives to damaged organs. The concept is based mainly on the manufacturing of bio-compatible scaffolds that may be designed to recapitulate the ECM of the original in terms of its cellular, biochemical, mechanical, and topographical properties [[60], [61], [62]]. Despite the detailed complexity, ECM could be seen to structurally exist with two phases, a fibril architecture and a hydrated interstitial gel. These two components' relative structural properties and functionalities vary drastically in different tissue types and can simplistically account for the diverse biomechanical properties of bulk tissues [14]. Thus, the achievement of these properties characterizing the actual tissue can be categorized into two distinct approaches based on the length scale, the non-fibril (gel) matrix and fibril architecture. They are frequently used as complementary to achieve functionally and structurally accurate models (Fig. 2).

Focusing on the fabrication feature sizes, Fig. 1 summarises the scale lengths at which the major biofabrication strategies operate. The cellular length scale approach uses biochemical and biological cues to bind cells and biomimetic matrices. Within the range of biofabrication, a combination of soft lithography, fibre spinning and hydrogel bioprinting cover the range of ECM components and cell sizes. By contrasting these length scales to some of the key features of the ECM, it is shown that cross-length scale biochemical and structural mimicry requires combining multiple fabrication techniques. Cells’ interactions with the external environment determine their behaviors, including migration, proliferation and apoptosis. Moreover, efforts of biofabrication at multiple length scales may hold the key towards functional, complex tissue models in the laboratory.

**Fig. 3:**
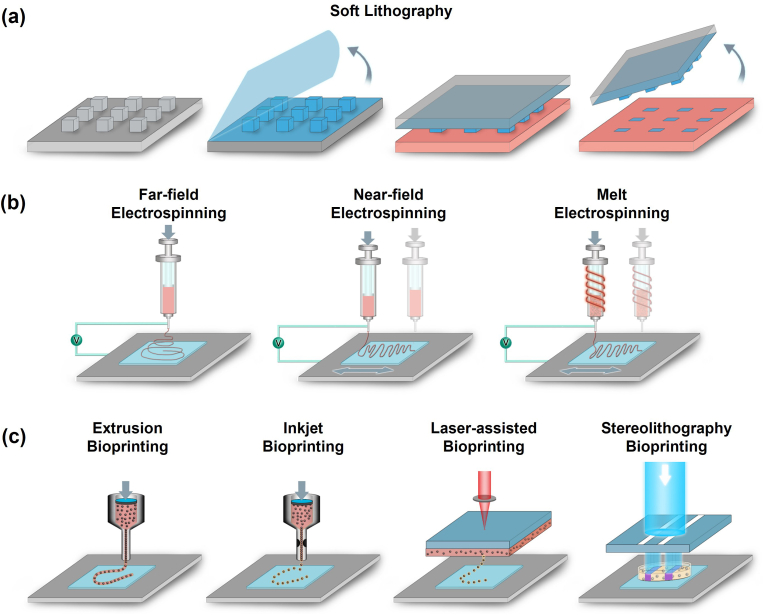
Different biofabrication approaches towards ECM-mimicking models. (a) Soft lithography. An elastomeric stamp is first coated with an extracellular matrix (ECM) material, which is then stamped onto a substrate surface to create micropatterns of the ECM material; (b) Schematic diagram of electrospinning techniques, including far-field electrospinning, near-field electrospinning and melt electrospinning; (c) Schematic illustrations of different hydrogel 3D-printing setups, including extrusion, inkjet, laser-assisted and stereolithography.

Consequently, rapid advances in tissue engineering and regenerative medicine showed the need to produce ECM‐mimicking biomaterials recapitulating the precise structural and topographical complexity and the porous and fibrous architecture [16]. While mimicking an incredibly complex matrix poses several issues and difficulties [75], the usage of soft-lithography (Fig. 3a), electrospinning (Fig. 3b), and 3D bioprinting (Fig. 3c) as strategies of *in vitro* modeling signals the beginning of generating functional mimicking scaffolds for regenerative medical attempts.

### 4.1 Soft-lithography microfluidics

Soft-lithography is a template-based microfabrication technique that semiconductors, optical storage media, and biomedical devices [[76], [77]]. It is often regarded as a technology with high reproducibility and high lateral resolution. Among these, polydimethylsiloxane (PDMS) is the most commonly used material, as it can make reversible conformal contact with substrates in complex geometries. Soft PDMS can generally replicate minimum features of >100 nm [78]. Leveraging its flexibility, PDMS has been molded to create diverse structures like pillars, valves, and stretchable membranes, which are further integrated into microfluidic devices [65,79].

**Fig. 4:**
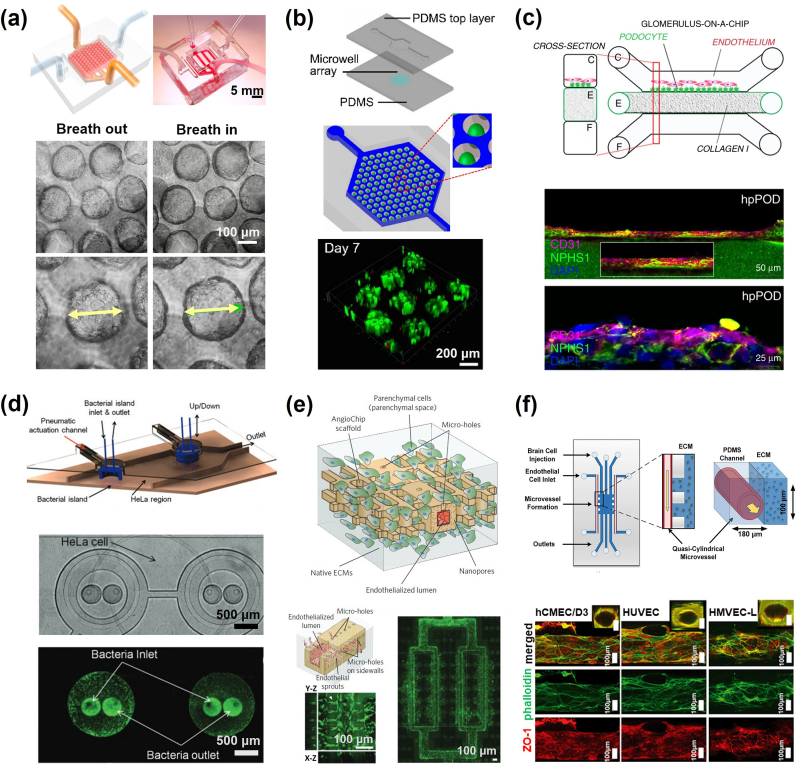
Demonstration of microfluidic organ-on-chip models with functionality in Tissue Engineering. (a) Schematic of an alveolar lung-on-a-chip model and micrographs showing the expansion of the sacs under a strain of 8% [87]; (b) Schematic of organ-on-a-chip and the confocal image of liver cancer microspheres cultured in 7 days [91]; (c) Seeding of podocytes and human glomerular endothelial cells in microfluidic chip with confocal images after 28 days [94]; (d) Intestine-on-chip model, establishing co-culture of HeLa cells and bacteria [95]; (e) biodegradable vasculature-on-chip model [101]; (f) On-chip perivascular niche with patient-derived glioma model [103]. (a) Reproduced from Ref. [87] with permission ofNational Academy of Sciences, ©2021; (b) Reproduced from Ref. [91] with permission of Springer Nature, ©202022; (c) Reproduced from Ref. [94] with permission of Springer Nature, ©2019; (d) Reproduced from Ref. [95] with permission of Royal Society of Chemistry, ©2010; (e) Reproduced from Ref. [101] with permission of Springer Nature, ©2018; (f) Reproduced from Ref. [103] with permission of Royal Society of Chemistry, ©2021.

Microfluidic devices are employed to cultivate cells and replicate the dynamic microenvironment experienced in tissues due microfluidic flows *in vivo* [[80], [81]]. Such advancement paved the way for organ-on-a-chip designs, where cells were integrated into microfluidic devices to simulate vital features of physiological microenvironment, including topography, mechanical cues, and metabolism [[82], [83], [84]]. Numerous organ-on-chip models that simulate diverse organ functions have been developed, including lung [,[85], [86], [87]], heart [65,88], liver [[89], [90], [91]], kidney [[92], [93], [94]], intestine [95,96], brain [97], vasculature [[98], [99], [100], [101]], skeletons [102], and tumor models [103] (Fig. 4). In the construction of organ-on-chip systems, several essential factors have been considered, such as the presence of chemical concentration gradients on demand, spatially defined cell culture, physiological cell-to-liquid ratio, microfluidic device-based shear force and mechanical stimulation, and environmental control of O_2_, CO_2_, pH, nutrients, and growth factors. Several key aspects have been considered in constructing organ-on-chip systems, including the on-demand presence of concentration gradients of chemicals, spatially defined cell culture, physiological cell-to-liquid ratio, microfluidic device-based shear force, and mechanical stimulation, as well as environmental control of O_2_, CO_2_, pH, nutrients, and growth factors [[104], [105], [106]]. Advances in organ-on-chip systems have also been examined from different aspects, including physiological function reconstruction [105,107], drug discovery [102,108,109], pharmacological research [110,111], disease modelling [112,113], immune reaction [114,115], stem cell and personalized medicine [116,117], cancer metastasis [118] and long-term biomedical investigation [119].

Additionally, soft-lithography offers substrates with topographic features such as grooves, pits, or posts, providing an approach for modelling the three-dimensional *in vivo* environment with fewer complications; surfaces with micron-scale topographic features have been shown to influence cell behaviors, including spreading, migration, differentiation as well as ECM deposition [[120], [121], [122], [123], [124]]. However, studies of this type have long been conducted with stereo-lithography to produce solid, rigid substrates which do not resemble the *in vitro* environment. Although great efforts have been exerted towards recapitulating the *in vivo* conditions of tissue/organs, the intrinsic shortfalls in organ-on-chip models are their lack of matrix diversity. Microfluidic substrates are physically and chemically different from ECM matrix while encapsulating fibrous matrix requires far more complicated fabrication processes. Hence, improving the matrix diversity in microfluidic devices still remains challenging, and only certain aspects of organs have been simulated from a physiological function or anatomic structure.

### 4.2 Electrospinning

Electrospinning is similar to other spinning techniques in that a tensile force extracts fibers from a spinnable source. Unlike traditional spinning techniques that employ physical mechanisms to initiate fiber formation, electrospinning harnesses the power of Coulomb's force from an external electric field to initiate the process [125]. In electrospinning, a phenomenon known as the ‘Taylor cone’ emerges in the droplet, and deforms the equilibrium where the droplet’s surface tension and the fluid’s viscosity offset the applied tensile force. This formation initiates the ejection of a fine polymer solution from the cone's apex towards the collecting substrate [126,127]. As the solvent of the polymer solution vaporizes along with the movement of the jet, a solid polymer fiber, with a diameter usually 100 nm or more, is collected in the end [[128], [129], [130]].

**Fig. 5:**
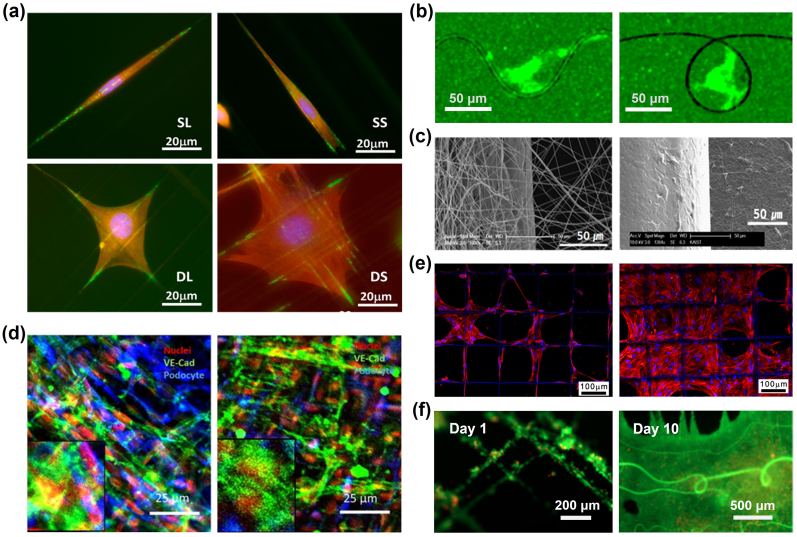
Demonstration of electrospun Nano/Micro-fibril structures with functionality in Tissue Engineering. (a) Fiber topography influences cell adhesion and morphology [140]; (b) Single cell behaviours differs in featured wavy and loop fibre patterns, as demonstrated in fluorescent channel [141]; (c) Internal fibrous microporosity creates an external coating of Chondrocytes on scaffold [145]; (d) Fibrous membrane creates two distinct but interacting tissue layers [146]; (e) Melt electrospun structure supports cell growth within large pores [147]; (f) Suspended fibers provide topographic guidance for fusion and expansion of cell aggregates over 10 days [148]; (a) Reproduced from Ref. [140] with permission of American Chemical Society, ©2014; (b) Reproduced from Ref. [141] with permission of IOP Publishing, ©2022; (c) Reproduced from Ref. [145] with permission of Elsevier, ©2008; (d) Reproduced from Ref. [146] with permission of Elsevier ©2018; (e) Reproduced from Ref. [147] with permission of IOP Publishing, ©2015; (f) Reproduced from Ref. [148] with permission of American Chemical Society, © 2022.

ECM mimicking electrospinning techniques have been continuously evolved over the past two decades [131,132]; and the applications have shown their ability in replicating similarities to the ECM of native tissues of several organs such as bone [133,134], skin [135], vascular grafts [136,137], cancer models [138], and tendon/ligament [139]. Electrospun fibers can also emulate the topography of the ECM fibrils; and as Nain and colleagues illustrated [140] (Fig. 5a), such topographies can directly impact cellular shape and the arrangement of focal adhesions. Zhang et al. furtherly applied low-voltage electrospinning to simplify ECM architectures into typical fiber patterns and conducted cell migration assays in response to well-defined fibril geometries [141] (Fig. 5b). Furthermore, specific surface patterns and structures of electrospun fibers have been proved crucial in directing and influencing the trajectory of cell migrations and consequently cell functions [142,143]. For instance, Xie et al. combined electrospun biodegradable polymers with embryonic stem cells, presenting a promising approach to improve nerve injury [144]. Through this method, they have successfully augmented the neural differentiation of mouse embryonic cells and facilitated neurite growth. Thus, cellular alignment is critical for overall cell functionality in tissues models, such as neural tissues and tendons, and can be used to direct appropriate stem cell differentiation.

The capacity to adjust the porosity and morphological configurations of electrospun fibers elucidates their paramount significance in diverse tissue engineering applications [[72], [149]]. Traditionally, far-field electrospun meshes offers small porosity (Fig. 5c) [145], which can be restrictive for cell infiltration and long-term viability [150,151]. Nonetheless, this feature can be leveraged for drug delivery applications and to replicate membrane structures inherent in the native ECM [152,153]. For example, Li et al. utilized electrospun patterns to create a dense fibrous co-culturing membrane device (podocytes and endothelial cells) and to model the functionality of the glomerulus in the kidney (Fig. 5d); the dense fibrous membrane enabled cross-talk and interaction between different cell types [[146], [154]]. Wang et al. fabricated microporous (5-6µm) electrospun polycaprolactone (PCL) vascular grafts with larger pores and thicker fibers, which notably improved cell infiltration and vascular regeneration [136]. Such grafts not only enabled complete endothelium coverage and efficient arterial regeneration, but also regulated macrophage behaviors, suggesting their potential as promising cell-free vascular graft alternatives for in-depth *in vivo* assessments. The porosity of fiber scaffolds has also been utilized to permit nutrient exchange whilst providing sufficient ECM structural compliance in response to physiologically relevant pulsatile flow conditions [155].

Integrating electrospun fibers into synthetic tissue constructs provides diverse biomechanical design alternatives [156]. A novel approach called focused rotary jet spinning (FRJS) was proposed to enable the rapid fabrication of micro/nanofiber scaffolds with programmable alignments in 3D geometries, and with this technology, the unique helically aligned structure of heart musculature was successfully constructed [157]. Visser et al. innovatively combined 3D-printed, high-porosity melt-electrospun PCL scaffolds with hydrogels, achieving biomechanical features that closely mirroring native articular cartilages [158]. This synergy not only enhanced the construct's stiffness significantly, but also promoted intensive cellular responsiveness under mechanical loading [159,160]. Electrospun fibers with outstanding mechanical properties produced by melt electrospinning have been employed as mesh mechanisms to trap cells, prompting the suspended cells to form organically structured tissue assemblies (Fig. 5e) [147,161]. To increase the role of the fibrous structure, Martine et al. reported that calcium triphosphate-coated electrospun scaffolds could guide primary osteoblastic cells to produce mineralized bone tissue and an osteoconductive environment [133]. More recently, the mechanical reinforcement provided by fibrous structures was utilized to enable *in vitro* measurement of the electrical properties of neurons grown in 3D [162].

Furthermore, electrospinning offers an alternative method to fabricate ECM fibril-mimicking fibrous-based substrates. These *in vitro* fibrous microenvironment allows scientists to engineer disease-specific models for topics including gastrulation [163], nonclosing chronic wounds [164], metastasis [165], and contributions of immune and inflammation [[166], [167], [168]]. To study the cellular interactions with different fiber surface microstructures, scientists have proposed an empirical model to quantitatively evaluate the formation of wrinkled, creased and porous fibre morphology from electrospinning [169]. In addition, advances in the fabrication of substrates with micron size have featured on a range of soft and hard substrates to identify parameters for *in vitro* tissue regeneration and *in vivo* implantations. For example, polymeric electrospun nanofibers have been widely used in neural tissue engineering for neural repair [[170], [171], [172]], regeneration guidance [170,173,174], Schwann cell maturations substrates [175] and neural stem cell differentiations [176]. Suspended fiber architectures in 3D cell culture has also been used as patterned fiber topography to guide the assembly of suspended high-cellular-density structures (Fig. 5f) [148]. However, related to this is the argument to utilize scaffold materials and methods that harness the innate regenerative capability of cells to build thick artificial tissues [177]. Scaffold architectures are crucial for directing cellular proliferation in high cellular concentrations, ensuring effective intercellular interactions without constraining future cell motility and growth [150,178]. At present, there is a scarcity of electrospinning methods that can effectively pattern electrospun constructs thicker than several millimeters, and this reinforces the motive to develop methods for patterning soft materials in 3D that allows deformation by cell remodeling processes.

### 4.3 3D bioprinting

Three-dimensional (3D) bioprinting emanates from additive manufacturing. It has become a new approach that aims to overcome the limitations of conventional 2D platforms and to build complex and functional tissue scaffolds with precise geometries and compositions [179,180]. 3D bioprinting strategies enable spatial manipulation of biomaterials and cells through layer-by-layer precise deposition [181] and allow the study of cellular interactions in 3D microenvironments [68,69]. Bioprinting introduces unique complexities compared to conventional 3D printing of plastics and metals. One notable challenge lies in the temporal dynamics of the process; bioprinted structures rapidly respond to internal and external cues, potentially inducing structural alterations post-deposition. These dynamic changes demand careful consideration of the process parameters and environment to ensure structural integrity and function. Another distinctive characteristic of 3D bioprinting pertains to the design process itself. During the design phase, substantial attention must be devoted to the scaffold's tailored mechanical and biological properties. The properties should allow for successful cell deposition and be conducive to restoring tissue and organ functions. Thus, the task is not simply constructing a physical structure; rather, it necessitates the creation of a biologically compatible environment that promotes cellular growth and functional integration [182,183]. Nevertheless, 3D bioprinting has gradually become a powerful tool for creating implants corresponding to patient-specific anatomy, more accurate phantoms for surgical planning, and disease models.

Commonly used 3D bioprinting approaches are extrusion, inkjet, laser-assisted, and stereolithography (SLA) methods [66,178,184,185]. These methods have the corresponding trade-offs between material tolerance and parameters such as printing speed, printing resolution, economic cost-effectiveness, cell loading density, etc [186]. The inkjet 3D bioprinting method is very similar to traditional 2D inkjet printing [182,187], in which the printer head connecting to the cartridge is deformed by a thermal/piezoelectric actuator, and droplets are generated with controllable sizes [181,186]. Similarly, laser-assisted printing utilizes a pulsed laser source to create high-pressure bubbles on the surface of ink layers and subsequently propelled droplets [67,188]. The falling ink droplet is then collected on the receiving substrate before being crosslinked/polymerized [66,189]. In comparison, extrusion-based bioprinting utilizes either air-pneumatic or piston for extruding hydrogels. It is the most commonly used printing technique, being compatible with a wide range of bioinks and high cell density loadings [181,182,190,191]. Besides, novel photo-lithography techniques provide an alternative approach [192,193], in which selective illumination of light-sensitive photoinitiators is used to generate free radicals and solidify inks in a layer-by-layer process [194], and to additively build up constructs [66,195].

**Fig. 6:**
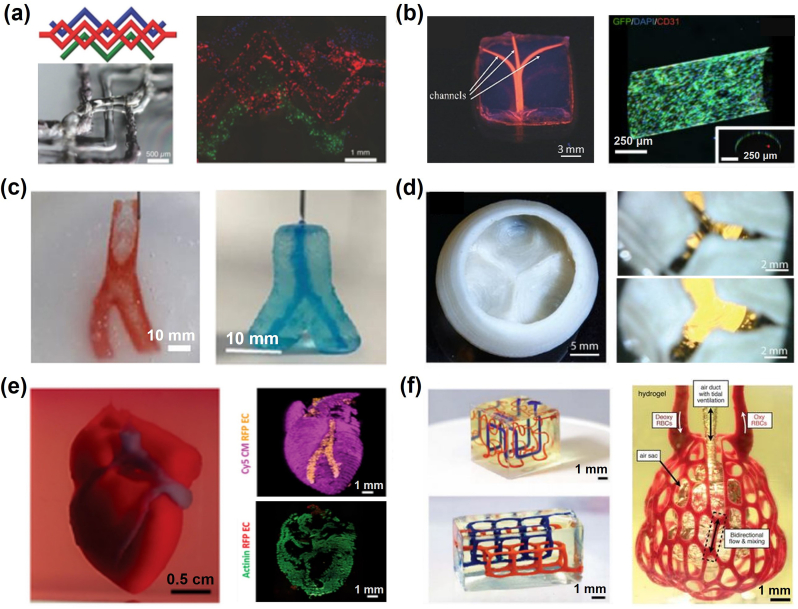
Demonstrations of 3D-bioprinted *in vitro* tissue/organ models with functionality in Tissue Engineering. (a) Schematics of vascular channel created using gelatin as sacrificial ink; fuorescence image of the printed vascular channel, with HUVECs (in red) and beads flow (in green) [202]; (b) photographs of the agarose-template created microchannels perfused with a fluorescent microbead suspension; longitudinal confocal images of a huvec-lined microchannel [210]; (c) Fabrication of a carotid artery structure using ‘SLAM’ strategy, demonstrating material durability and perfusion [211]; (d) Top view of FRESH-printed collagen heart valve using ‘FRESH’ strategy, and a sequence of valve opening under pulsatile flow over ∼1s [206]; (e) A printed heart within a support bath; 3D confocal image of the printed heart (CMs in pink, ECs in orange; sarcomeric actinin in green); [215]; (f) Entangled vascular networks with mathematical space-filling curves to entangled vessel topologies within hydrogels; Photograph of a printed hydrogel containing the distal lung subunit [216]. (a) Reproduced from Ref. [207] with permission of Wiley-VCH, ©2019; (b) Reproduced from Ref. [210] with permission of Royal Society of Chemistry, ©2014; (c) Reproduced from Ref. [211] with permission of Wiley-VCH, ©2019; (d) Reproduced from Ref. [206] with permission of American Association for the Advancement of Science, ©2019; (e) Reproduced from Ref. [215] with permission of Wiley-VCH, ©2019; (f) Reproduced from Ref. [216] with permission of American Association for the Advancement of Science, ©2019.

Several techniques have emerged from these four bioprinting methods. For instance, 3D Embedded printing, a variant of microextrusion-based printing technology, has expanded the possibilities across various fields. It enables printing inks that were previously challenging to use [[196], [197], [198]]. In particular, sacrificial support inks, a subset of embedded printing techniques, has facilitated the creation of hollow cavities and intricate vasculature networks within hydrogel matrices [100,[199], [200], [201], [202], [203]]. For instance, Lewis's group has been widely using Pluronic F-127 as an aqueous fugitive ink (Fig. 6a) for embedded printing of 3D vascular networks; the sacrificial ink could be fused away by cooling the temperature below 4 °C [202]. Based on their diffusing mechanisms, sacrificial supporting inks can be divided into water-dissolving inks (carbohydrate glass [199,204]), thermo-transition inks (Pluronic F-127 [[200], [201], [202],^205^], gelatin [[206], [207], [208], [209]] and agarose [210,211]), and chemical-transition inks (alginate [212,213]). Furtherly, researchers have evaluated the “shapeability” of distinct hydrogel inks and diverse support baths; it is indicated that the dominate mechanisms for interfacial instabilities, including diffusion-driven or charge-driven, can be predicted by evaluating the composition pairing of the pre-crosslinked hydrogel ink and supportive bath [214].

Gelatin and agarose gelation properties attracted more researchers to use these materials to recreate tubular hollowed channels (Fig. 6b). Following the concept of embedded extrusion printing, an agarose-microparticle supporting bath was introduced in the ‘Suspended Layer Additive Manufacturing (SLAM)’ strategy for printing tubular geometries from low-viscosity solutions [211] (Fig. 6c). An agarose-based fluid gel could also suspend the printing object in its liquid form before the gelation and extraction from the printing bath. Thomas J. Hinton et al. applied thermo-reversible gelatin-microparticle slurry as a sacrificial supporting bath; a ‘Freeform Reversible Embedding of Suspended Hydrogels (FRESH)’ printing strategy was developed to build up 3D aortic valve scaffolds with multiple ECM-mimicking materials including alginate, fibrin and collagen [206,209] (Fig. 6d).

In another example, research teams from 10.13039/501100004375Tel Aviv University characterized a Xanthan-gum-formulated fugitive ink to support the printing of volumetric scaffolds with ECM-mimicking materials. Such Xanthan gum-based supporting ink can support prolonged, precise printing (resolution ∼10 μm) for at least 18 h; thick and perfusable cardiac patches and miniature heart models were printed as demonstrations [217,215] (Fig. 6e). In comparison, recently, a photolithography-based printing strategy was addressed as ‘Stereolithographic Apparatus for Tissue Engineering (SLATE)’ [216] (Fig. 6f). This strategy applied a set of novel biocompatible photo-absorbers for high-fidelity embedded-printing of multivascular networks inside PEGDA and GelMA hydrogels; complex multivascular networks can be generated. Such vasculature-embedded constructs could be used for functional albumin-producing hepatic models.

Compared to previously described methods, 3D bioprinting is a versatile technique in biofabrication, particularly in assembling volumetric tissue/organ scaffolds [218]. This is based on its major advantages in a vast material library, design flexibility, rapid prototyping, and multi-material printing ability [178,219]. Advances in bioprinting techniques provide accessible platforms for replicating the biochemical and mechanical properties of the ECM microenvironment, hence providing a matrix for cell encapsulation, cell binding, and a reservoir for growth factor release [220,221]. Bioprinted cell-laden 3D scaffolds provide spatial depth and better cell-cell communication for improved *in vivo* physiology [222,223]; they can also be supplementary to animal tests and contribute to predicting human toxicological and pathophysiological responses [224,225]. However, 3D bioprinted organ models remain in the stage of small-scale application, and existing studies reported some major challenges inducing low production accuracy [226], limited simulation characteristics [227], and long-term cell viability and functionalities [228,229].

### 4.4 Self-assembly

Self-assembly is referred to as the automated aggregation of individual small molecules into well-defined and reproducible hierarchical structures using non-covalent interactions such as van der Waals, hydrogen, hydrophobic, and electrostatic forces [230]. Self-assembly has functionally evolved in nature from individual proteins/DNA molecules’ folding to higher-order assemblies of phospholipids into cellular membranes [231]. Besides, in the context of Tissue Engineering and Regenerative Medicine, self-assembly offers unparalleled advantages in unprecedented programmability and innovative capacity to interact with cells with high selectivity [[232], [233]]. Through this approach, functional nanomaterials have been developed using peptides [[234], [235], [236], [237]], proteins [238], DNA [239,240], and polymers [241], among others [242,243]. These self-assembly approaches also enable enhanced biomimicry, molecular versatility, cell communication, and overall bioactivity.

However, there are also important challenges for self-assembly to overcome, such as the capacity to self-assemble immediately robust structures, high costs, and scalability constraints. Nonetheless, given the need to better recreate the distinctive molecular, structural, and cellular complexity of biology, it is envisaged that self-assembly will continue to be integrated with biofabrication through both emerging self-assembling platforms as well as enhanced printing methods. For example, the ability to print within complex environments enabling simultaneous extrusion and growth of self-assembling structures [244] could significantly enhance resolution, bioactivity, and level of biomimicry. Another important step will likely come from improved self-assembling systems that enhance structural integrity, for example, through the addition of host-guest interactions [245], modulation of mechanical properties via interactions between different components through non-covalent [246], or covalent co-assembling processes [247].

## 5 Recent applications of the biofabrication of ECM-like substrates for different organ culture models

**Fig. 7:**
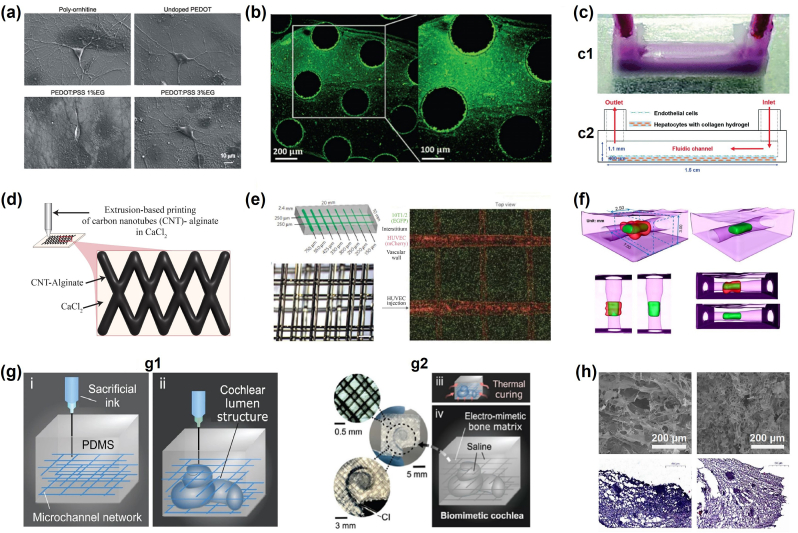
Applications of the biofabrication of ECM-like substrates for organ and cancer models: (a) High magnification micrographs show the healthy morphology of single neurons grown on the different substrates including poly-ornithine, PEDOT:PSS 1% ethylene glycol (EG) and PEDOT:PSS 3% EG Ref. [252]; (b) Fluorescence images showing fluorescein-HA (green) on the HA-binding peptide (HS-Pep-1) printed areas [253]; (c) Schematic of (c1) the one-step bioprinting method of the liver-on-a-chip model and (c2) a side view of the live-on-a-chip model [254]; (d) Illustration of 3D-bioprinted hybrid implant made of CNT-incorporated alginate and photo-cross-linked cell-laden hydrogel at 5 μg CNT/mg hydrogel [255]; (e) Schematics of vascular channel created using gelatin as sacrificial ink; fluorescence image of the printed vascular channel, with HUVECs (in red) and beads flow (in green) [199]; (f) Micro-CT scanning demonstrating a merged 3-D image of reconstructed open lumen construct in a thick collagen scaffold [207]; (g) Schematics of the embedded 3D printing strategy to produce the electro-mimetic bone matrices and the biomimetic cochleae (g1 and g2) [256]; (h) Scanning electron microscopy imaging of bioinks (comprised of hyaluronic acid, sodium alginate and gelatin), as well as Hematoxylin–eosin staining of Human glial cells within such bioinks [257]. (a) Reproduced from Ref. [252] with permission of Frontiers Media S.A., ©2015; (b) Reproduced from Ref. [253] with permission of Royal Society of Chemistry, ©2019; (c) Reproduced from Ref. [254] with permission of Royal Society of Chemistry, ©2016; (e) Reproduced from Ref. [199] with permission of Springer Nature, ©2012; (f) Reproduced from Ref. [207] with permission of Elsevier, ©2012 (g) Reproduced from Ref. [256] with permission of Springer Nature, ©2021; (h) Reproduced from Ref. [257] with permission of Springer Singapore, ©2020.

The biofabrication of an ECM-like material is primarily determined by the composition of the intended *in vivo* ECM. Living tissues have distinct functions and structures, necessitating the implementation of distinct ECM properties. Additionally, the intended application of the constructed model plays a crucial role in determining the extent of ECM mimicry [248]. *In vitro* models, such as microfluidic and organ-on-chip models, tend to focus on mimicking the cell niche in order to produce *in vivo*-like functions [248]. On a larger length scale, such as scaffolds used in regenerative medicine and implants, the anatomy and additional physical properties, such as mechanical and electrical properties, receive more attention and have a higher impact on the model's efficiency [[249], [250], [251]]. In the following section, we will discuss different applications of the development of ECM-like models (Fig. 7) which target different physical aspects of *in vivo* ECM and thus require distinct materials and biofabrication approaches (Table 2).

**Table 2:** Applications of the biofabrication of ECM-like substrates.

Organ	Application	Biomaterials	Biofabrication method	Important findings	References
**Brain**	*In vitro* neural wiring model	GelMA	Extrusion-based Bioprinting	• Development of a reproducible method for differentiation of hiPSCs in highly pure sensory neurons (SN) • SN populations • confirmed to be able to detect external stimuli	[285]
	Neural stem cell transplantation	PEDOT:PSS with GelMA	Multistep fabrication method based on Top-down approach	• The model showed to enhance neuronal activity • Lower inflammatory responses in *in vivo* model of middle cerebral artery occlusion	[286]
**Blood-brain barrier**	BBB-on-a-chip model	Collagen-matrigel	Multistep fabrication method based on Top-down approach	• Promotion of cell growth of various types of cells • Promotion of the adhesion-associated intracellular signalling	[287]
	*In vitro* BBB model	PEG	Multistep fabrication method based on the top-down approach	• Low elastic modulus • Tunable mechanical properties of the substrate	[288]
**Liver**	*In vitro* liver model recapitulating native metabolic zonation	dECM	Freeze drying method	• Minimal thrombogenicity • Reduced foreign-body response • Enhanced pro-remodeling macrophage activation	[289]
	Liver-on-a-chip model	Collagen	Extrusion-based bioprinting	• Biofabrication of a liver-on-a-chip model using a one-step bioprinting process • Achieving spatial heterogeneity and complexity	[290]
**Heart**	Cardiac patches	Methacrylated type I collagen + carbon nanotubes	Micropatterning and extrusion-based bioprinting	• High viscoelasticity and electrical conductivity • Obtention of higher cellular proliferation, migration and differentiation	[291]
	Myocardial scaffold	rGO-incorporated GelMa	Multistep fabrication method based on Top-down approach	• Better cell viability, proliferation and maturation • Stronger contractility and faster spontaneous beating rate	[292]
**Vascula-ture**	Vascularized scaffod	Agarose/Alginate/Fibrin/Matrigel	Casting	• Achievement of perfusable vascular networks • The vascular channels can sustain metabolic function of primary rat hepatocytes	[199]
	Multi-scale vascular network	Gelatin	Extrusion-based bioprinting	• Construct larger fluidic vascular channels with a diameter of 1 mm • Enabling the formation of adjacent capillary networks through natural maturation	[208]
**Bone**	Bone scaffold	Collagen-apatire nanocomposite	Bottom-up crystallization method	• High regeneration properties and vascularization • Extracellular matrix Secretion of the ECM • Mineralization of rat bone marrow stem cells	[293]
	Cochlear implant	Electro-mimetic bone matrices	Extrusion-based bioprinting	• Achieving a tuneable electro-anatomy of the matrix • Developing a co-modeling approach based on 3D printing and machine learning • Predicting the cochlear tissue resistivity	[294]

### 5.1 Brain

The brain ECM accounts for approximately 20% of the brain volume [295]. It mainly comprises glycosaminoglycans, proteoglycans, glycoproteins, and fibrous proteins, with only a trace of type IV collagen [296]. The brain has a low elastic modulus ranging from 0.1 to 1 kPa, which can be explained by the absence of type I collagen, which typically forms stiffer and longer fibers [297,298]. This specific ECM is composed of three main parts, including perineuronal nets (PNNs), the basement membrane (BM), and the interstitial matrix (ISM) [299]. The PNNs are dense specialized structures surrounding specific neuronal cell types mainly responsible for synaptic stabilization in the adult brain [300]. The basement membrane is a thin layer of extracellular matrix (ECM) that faces the basal side of brain endothelial cells and plays an essential role in maintaining the blood-brain barrier [301]. The interstitial matrix is a connective tissue between the central nervous system and the parenchyma. It consists of a dense network of proteoglycans, hyaluronan, tenascins, link proteins, and relatively small amounts of fibrous proteins and adhesive glycoproteins [302].

The brain ECM plays a crucial role in the nervous system, such as assuring cell-cell and cell-matrix communication and regulating the proliferation, migration, and synaptic integration of neurons and neuroglia [303]. Minor changes to its mechanical and chemical properties can impair brain function. Multiple brain pathologies, including Alzheimer's disease, epilepsy, and strokes, have been linked to these ECM-changing properties [304]. *In vitro* studies, for example, revealed that ECM stiffness was related to cell phenotypes [223,305]. Furthermore, the anisotropy of the brain ECM, which dictates cell arrangement, plays a pivotal role in mediating intrinsic cell interactions [306]. The anisotropy is commonly engineered by modifying the substrate's topography or material composition. Regarding substrate topography, various designs, such as grooves, ridges, filaments, fibers, and composite arrangements, have been utilized. These designs have demonstrated an ability to provide adequate anisotropy across different scales [[307], [308], [309]]. As for the material composition, both natural and synthetic materials have been employed to mimic the brain extracellular matrix (ECM). Natural polymers used for this purpose include collagen, gelatin, hyaluronic acid, agarose, alginate, fibrin, and chitosan [257,[310], [311], [312]]. The synthetic materials include polycaprolactone, poly-l-Lactic acid, poly-D, L-lactic-co-glycolic acid, and conductive polymers such as polypyrrole (PPy) [[285], [310], [311], [312]].

It is well established that the brain's electrical activity is crucial for its functioning and development. Several studies have focused on designing new conductive polymers for either *in vitro* or *in vivo* applications [313]. Various conductive polymers, including polyaniline, PPy, and poly(3,4-ethylenedioxythiophene) (PEDOT), are increasingly regarded as promising biocompatible polymers which are capable of mimicking the electroconductivity of biological tissues [313]. A mixture of the two ionomers, PEDOT and polystyrene sulfonate (PSS), for example, has been shown to generate a favorable microenvironment that stimulates neuronal growth and synapse formation (Fig. 7a) [252]. Combining PEDOT:PSS with GelMA has offered Zhang et al. a better alternative [286]. Their research study focused on the use of this hybrid hydrogel to treat ischemia-reperfusion injury. Indeed, this combination enabled the researchers to create a conductive polymer with a high-water content that regulates neural stem cell development and improves the anti-inflammatory effects of neural stem cells on ischemia-reperfusion-injured tissues.

Besides the material physical properties of the biofabricated brain ECM, its porosity is extensively considered to improve cell migration and nutrient exchange [314]. The research study led by Tang-Schomer et al. confirms the correlation between substrate porosity and brain model accuracy [315]. Their work described the design of an ECM-like composite using a silk-protein-based porous scaffold, which supported primary cortical neurons. The proposed modular brain-like cortical model has offered a robust structure that allows the exhibition of *in vivo*-like functional and electrophysiological activities.

Bioprinting methods have facilitated the biofabrication of artificial brain ECM and neural tissues [316]. They have also permitted the achievement of comparably complex structures [317]. Loranzo et al., in a fascinating study, have designed a peptide-modified polymer, the gellan gum-RGD (RGD-GG), that encouraged cell proliferation and neural network formation but also facilitated the construction of a multilayer that has the potential of mimicking the *in vivo* cerebral cortex layers [318]. Currently, there are a plethora of bioprintable materials that have also been demonstrated to have the ability to improve the 3D neural tissue models further [26].

### 5.2 Blood-brain barrier

The blood-brain barrier (BBB) is a selective barrier in the human brain which protects neural tissue from toxic substances [319]. The BBB is covered with a basement membrane of two layers from the brain endothelial cell's basal side and glycocalyx from the laminal side of these cells [301]. Mimicking the ECM of the BBB differs from other organs' ECM by the challenge of making an extremely thin 2D layer instead of a 3D structure. This thin layer, 20–200 nm, should also have a very limited permeability [320].

Various strategies for imitating the BM of the BBB have been developed [321]. Most of these solutions emphasize lowering the ECM-like layer's thickness more than other characteristics, such as its biomechanical, biochemical properties and electrical properties. Porous membranes, Electrospun substrates, and ultrathin membranes have been widely used to achieve *in vitro* BBB with acceptable permeabilities [322]. On the other hand, hydrogels offered accurate stiffness and viscoelasticity to the native ECM; however, current biofabrication methods could not achieve that kind of ultrathin layers [323]. This application has also used synthetic, natural, and dECM [[287], [288], [323]].

Contrary to the BM, glycocalyx has been commonly neglected, and only a few tentatives of recreating a similar layer to improve the accuracy of biofabricated BBB or vascular models have been reported [322]. This thin layer is found to be very rich in hyaluronic acid. Therefore, mimicking the glycocalyx (GCX) using HA is the easiest and most opted solution (Fig. 7b) [253].

The vasculature's ECM is generally simplified to a 2D planar substrate. However, recent research studies have proved the importance of providing a curved environment to regulate cell behavior [324,325]. To achieve that, several research groups developed more advanced approaches that allow the recreation of the vasculature's curved geometry. Microfabrication methods such as needle modeling, which uses a needle as a mold to create a hollow hydrogel structure, and finger patterning, which uses the gradient of viscosities between two fluids to create hollowed structures, have piqued the interest of researchers in the field and contributed to the development of more accurate BBB and vascular models [326]. Unfortunately, the required precision to achieve *in vitro* BBB with low permeability is currently limiting the application of bioprinting methods in this field.

The development and design of suitable ECM for *in vitro* BBB model are assessed by the two main characteristics, including permeability and the Transepithelial/transendothelial electrical resistance (TEER) measurement. TEER represents the ionic resistance of the blood-brain barrier. The higher the measured TEER value, the less permeable the model will be. However, available blood-brain barrier models still regard TEER measurement as unprecise. Therefore, using conductive polymers is considered a promising approach to not only facilitate the assessment of the barrier but also provide a better mechanical and electrical environment for neurons and glial cells to create even more comprehensive models such as neurovascular and central nervous system models [327].

### 5.3 Liver

The hepatic ECM represents only 10% of the volume of a liver [328]. It is mainly distributed between the endothelial and epithelial cells [328]. Despite its low abundance, the hepatic ECM is essential for maintaining and guiding hepatocyte differentiation. The hepatic ECM mainly comprises fibronectin and small quantities of collagen type I, III, IV, V, and VI [329]. Biofabricated polymers have been widely used to recreate the 3D hepatic structure and cell-cell interaction while preserving essential hepatic functions [330]. However, the limited cell-matrix interactions, polarity, and durability are a big challenge that hander the achievement of an *in vivo*-like albumin secretion and urea synthesis [291].

Liver zonation is a crucial characteristic of the liver that refers to the segregation of hepatocytes into three zones: periportal, central lobular, and perivenous zones [331]. This separation is based on the gradients of different components and functions in the liver, such as oxygen, glycogenesis, and glycolysis. Several studies tried to recapitulate this zonation *in vitro* using PDMS microfluidics, which failed to assure long-term functional stability and continuous perfusion [289]. Remarkably, a combination of decellularized liver-ECM was used in a recent study to host primary neonatal rat hepatocytes to mimic the liver zonation [290]. As a result, the ECM-like scaffold offered long-term functional stability over 45 days by recapitulating the liver zonation.

Feasibility is another critical parameter that is primarily considered during the design of ECM-like materials. As described before, bioprinting methods are versatile and advantageous when it comes to freeform 3D structures. Therefore, by considering the liver's structural complexity, it is natural to choose bioprinting methods to achieve an *in vivo*-like liver structure. For instance, HepG2 and HUVEC are encapsulated in type I collagen and gelatin hydrogels to construct a liver-on-a-chip model that uses PCL as the structure of the model using a one-step extrusion-based bioprinting method (Fig. 7c) [254]. The liver-on-a-chip model has mimicked the dynamic conditions which helped stretch endothelial (similar to what happens *in vivo*) and, simultaneously, 3D ECM-like tissue structure that cannot be achieved using other biofabrication approaches.

### 5.4 Heart

Cardiac ECM is characterized by its highly complex and dynamic structure [332]. Cardiovascular ECM, like other ECM, is in charge of organ homeostasis and mechanical support. It comprises a basement membrane containing mainly laminins, type IV collagen, and proteoglycans and an interstitial matrix composed mainly of type I and III collagens [333].

Polyethylene glycol and other synthetic polymers have been widely employed to generate a cell-friendly microenvironment for cardiac tissue [291]. They have also been mixed with decellularized ECM to create hybrid structures, which have been shown to be more promising and efficient [291]. This mixture expanded the potential of both synthetic polymers, mainly PEG and dECM, which allowed the tunability of the mechanical properties and the encapsulation of cells for a comparably long period, five days. Natural polymers such as collagen, gelatin, and alginate have previously suffered from low mechanical properties and are regarded as mechanically limited scaffolds compared to the *in vivo* cardiac ECM [292]. However, their mixture with photopolymerizable materials has improved the construct stiffness [255]. For example, methacrylate has been mixed with type I collagen to create the methacrylated type I collagen (MeCol), which was bioprinted alongside carbon nanotubes (CNTs) to obtain a conductive and resilient structure (Fig. 7d) [255]. Gelatin methacryloyl (GelMA) is another example of photopolymerizable mixed polymers. This particular polymer is utilized extensively in bioprinting and is modifiable, which justifies its selection as a scaffold material for cardiac tissue. Additionally, modifications such as the incorporation of reduced graphene oxide (rGO) in GelMA have improved the biocompatibility of the hydrogel, cell viability, proliferation and maturation, cardiomyocytes contractility, and marked faster beating rate [334].

The mixture of multiple materials, including polymers and polymer composites, usually called hybrid materials, has great potential to overcome physical and chemical limitations to create a more suitable environment for cardiac tissues [335]. Notably, the addition of nanoparticles and microfibers provided favorable conditions for cell proliferation and obtention of ECM-like mechanical properties. For instance, Zhang et al. used a hybrid approach that uses extrusion-based bioprinting and a dual-step crosslinking procedure to produce a vascularized cardiac *in vitro* model [336]. Electrical conductivity is another important physical property for creating accurate *in vivo*-like ECMs. Besides rGO, metallic nanoparticles such as gold nanoparticles have been incorporated into biopolymers to enhance electrical conductivity [337]. Additionally, the research study led by Baei et al. proved that combining gold nanoparticles with chitosan also has excellent potential for maintaining cell viability and growth rate [337].

Cardiac tissue alignment significantly hinders mimicking the heart's ECM, which is required to accurately replicate heart function and pathology for drug development and regenerative repair. Currently, there are two approaches to constructing aligned cardiac tissue models: the cell seeding approach based on seeding cardiomyocytes onto biofabricated substrates and the bioprinting-based approach. On the one hand, the cell seeding approach is the most common approach that relies on microfabrication methods such as electrospinning and 3D printing to create aligned patterns that will later serve as a guide to direct the seeded cardiomyocytes [[338], [339], [340]]. The main challenge in this approach is the accuracy of the microfabrication method used to create these patterns, which has pushed research teams to develop more sophisticated methods allowing precise control of the aligned substrate, such as the previously described FRJS method. For instance, in a recent study, Cheesbrough et al. used electrospun nanofiber sheets from polyester-based-polyurethane-urea-polyhedral-oligomeric silsesquioxane elastomer (P(EDS)UU-POSS) to culture skeletal myocytes and thus created skeletal myofibers [341]. The model showed excellent mechanical properties (Young modulus of 25 kPa) and high cell viability, attachment, alignment, and differentiation.

On the other hand, another bioprinting-based approach is a bottom-up approach that simultaneously deposits cells with their substrate in a mixture referred to as bioink. Ahrens et al. have employed this approach by first creating anisotropic organ building blocks (aOBBs) that were later bioprinted to create cardiac tissue with high cellular density [342]. The alignment was induced using shear and extensional forces, usually generated in extrusion-based bioprinting when tapered nozzles are used. As a result, the model demonstrated high contractile functions with changeable magnitude and direction. Finally, both approaches presented good structural mimicry of the *in vivo* cardiac tissue with promising applications on different scales. However, more development is needed to create ECM-like materials that imitate both mechanical and electrical properties at the same time.

### 5.5 Vasculature

Incorporating vasculature remains a crucial challenge in biofabricating *in vitro* tissue and organ models. Constructing vessels at various length scales and widespread 3D structures is incredibly demanding. This importance stems from vasculature's essential role in supplying natural tissues with nutrients, oxygen, and other vital components necessary for tissue development and sustenance [343,344]. The composition of the vascular extracellular matrix (ECM) varies across each layer of the vasculature, and it's noteworthy for its ability to promote the formation of complex three-dimensional (3D) networks while also providing an elastic and adaptable microenvironment. Several biofabrication methods have been employed to replicate the structure of the vasculature. However, so far, none have been successful in recreating its ubiquitous tubular 3D structure.

In addressing these challenges, substantial progress has been made using embedded bioprinting techniques, leveraging a wide variety of ink and support bath materials. These advancements showcase the evolving versatility of this approach in overcoming the intricacies of the fabrication of complex vascularized models. In 2012, Miller et al. first reported tubular networks being produced in a hydrogel matrix with log-pile arrays of carbohydrate glass [199] (Fig. 7e). Later on, Gelber et al. extended the capabilities of sugar-alcohol printing by combining carbohydrate glass with a modified 3D printer and achieved the printing of intricate free-form structures [204]. Similarly, due to their thermo-reversibility, researchers have used solidified gelatin and agarose as alternative supporting templates to construct perfused vascular channels within the collagen matrix (Fig. 7f). After the collagen got crosslinked, the embedded gelatin can be reversibly liquefied at 37 °C and leached out, leaving the hollow vascular channels [207,208]. With embedded-printed vascular networks, printed hydrogel constructs could be constantly perfused and resided with biologically relevant matrixes for building desired *in vitro* tissue models.

In summary, biofabricating accurate vasculature for *in vitro* tissue and organ models is challenging due to the inherent complexity of its 3D structure. However, significant advancements have been made using embedded bioprinting techniques, utilizing diverse materials and thermo-reversible substances. Additionally, ECM-mimicking models have enabled progress in drug screening and disease modeling. Innovations such as the SLAM and FRESH strategies and Xanthan-gum-formulated fugitive ink show promising potential for high-precision bioprinting. Despite challenges, these developments highlight the promising future of vasculature replication in biofabrication.

### 5.6 Bone

The bone is mainly composed of ECM inorganic compounds (60%) and organic compounds (40%) [345,346]. Unlike the brain ECM, the bone matrix is 90% type I collagen, which explains the stiffness of this type of connective tissue. Besides type I collagen, type III collagen, type V collagen, proteoglycans (PGs), biglycan, and chondroitin sulfate (CS) chains are also present in the bone matrix and together are essential to obtain proper bone toughness [347]. Unlike other tissues where the softness of the substrate plays a major role in assuring the organ's proper functioning, toughness, by contrast, is the most important parameter in engineering bone ECM-like materials. Designing structures that resist high mechanical stress and simultaneously assure cell-cell, cell-matrix interactions, osteoinduction, osteoconduction, and osteointegration is extremely challenging. Previously mentioned polymers, such as hyaluronic acid (HA) and type I collagen, cannot meet the chemical and mechanical properties required to replicate bone tissue. As such, they are often utilized as a coating for more rigid materials like metals [348]. For instance, Sartori et al. designed a titanium implant that was coated using type I collagen [294]. The research found that non-coated and coated implants have both allowed bone ingrowth. However, the titanium-coated implant showed a more significant osteointegration.

Bioceramics are another solution for bone regeneration and, more specifically, beta-tricalcium phosphate (β-TCP), the most used scaffold in this field [293]. The β-TCP has both osteoinductive and osteotransductive potential due to its very similar solubility to bone minerals, making it resorbable by osteoclasts [293]. However, Liu et al. considered the lack of flexibility of β-TCP and its slow degradation as a major drawback which pushed the research team to create a microstructured composite containing calcium phosphate microparticles and biodegradable polymers such as collagen [349]. The nanocomposite collagen-apatite (Col-ap) has indeed offered a bone-like nanocomposite structure, activating bone ingrowth and promoting its vascularization.

Similar to previously detailed applications of conductive polymer, bone is yet another biological tissue whose electrical properties have commonly been ignored in *in vitro* and *in vivo* models. Interestingly, Lie et al. targeted this limitation and constructed a cochlear implant that mimics the structure of the *in vivo* cochlea and its electrical conductivity (Fig. 7g) [256]. The research team developed a combined approach based on neural network machine learning and 3D printing, which allowed them to predict the stimulation performance of the designed cochlear implant and thus construct patient-specific accurate implants.

Although these efforts to create *in vivo*-like structures for the *in vitro* bone modeling or grafts, these artificial materials still fail to replicate the objective complexity. Decellularized bone ECM has been regarded as a more promising alternative. In point of fact, they have the potential to provide low immunogenicity while retaining the tissue's original mechanical properties [350].

### 5.7 Cancer models

Tumor cells begin to proliferate in the ECM of the host tissue [351]. As a result, the ECM undergoes several biophysical and biochemical processes induced by remodeling mechanisms, including ECM deposition, chemical modification at the post-translational level, proteolytic degradation, and force-mediated physical modelling [352]. These changes affect different aspects of the tumor development and induce pro-tumor signaling, which causes alterations of the ECM physical properties [[352], [353]]. For example, the expression of type I collagen was reported to be significantly higher in liver, lung, breast, and metastatic ovarian cancers [354]. Other ECM components, including hyaluronic acid, fibronectin, and laminin-332, were also frequently found to be elevated [355,356].

The biofabrication of *in vivo* mimicking ECM structure mainly depends on the original tumor site. For instance, the brain ECM mainly comprises Hyaluronic acid (HA). Therefore, Ma et al. chose HA as the base for their bioink. The research team has used an extrusion-based bioprinting approach to construct the brain ECM mimetic microenvironment model (Fig. 7h) [257]. Interestingly, the model enabled researchers to precisely mimic the *in vivo* glioblastoma stroma environment. In another study, GelMA was considered another option to recreate the GBM environment since it provides good biocompatibility and shear-thinning properties [357]. As a result, the model successfully displayed macrophage recruitment, polarization, and GBM progression. Therefore, the recapitulation of the components of the *in vivo* tumor microenvironment is important and its suitability with currently advanced biofabrication methods such as bioprinting. For example, Mazzaaglia et al. created a novel bioprinting method employing a custom-designed printhead and a modifiable robotic arm for printing compartmentalized tumoroids [358]. These tumoroids incorporate cancer-associated fibroblasts (CAFs) and closely resemble *in vivo* structures.

Biofabricated 3D cancer models are better for mimicking tumor microenvironments [293]. As a natural outcome, multiple research teams have been able to test therapies and anticancer drugs for various forms of cancer using these models. Wang et al., for example, used extrusion-based bioprinting to create a 3D breast cancer model [359]. The model had a mixture of methacrylated HA, methacrylated gelatin in addition to non-modified HA and gelatin to ensure a biocompatible microenvironment for adipose-derived mesenchymal stem cells (ADMSC) and human epidermal receptor 2 positive breast primary cancer cells (21 PT). It has served to test treatment with a lysyl oxidase (LOX) inhibitor, which caused a decrease in LOX secretion and downregulated adenosine triphosphate-binding cassette transporter gene expression. The same study compared 2D and 3D breast cancer models, showing that 3D bioprinted models were far more reliable for drug testing and screening.

Finally, ECM-mimicking tissue models are useful platforms to facilitate systematic investigations in drug screening and disease models [344,360,361]. Recent advances in biofabrication techniques have demonstrated prospective capability in replicating mechanical and physiological features of the ECM, as well as constructing biomimetic *in vitro* models [[362], [363], [364], [365]].

## 6 Conclusion and future perspectives

As our understanding of the ECM microenvironment continuously evolves, the development of ECM-mimicking models has been proved to hold great potential in tissue engineering and regenerative medicine. ECMs are highly heterogeneous architectures with organ-specific morphological, physical, chemical, and biological properties. In this review, we highlight important ECM features and functions in the context of tissue engineering and regenerative medicine, and we investigate different ECM-mimicking materials, including natural polymers, synthetic polymers, and decellularized ECM tissues. We also examine different techniques, such as soft lithography, electrospinning and 3D bioprinting, for their potential to preserve tissue-specific structures and for the purpose of designing matrix-mimicking scaffolds for translational applications. Finally, we compare the opportunities and challenges of various ECM-mimicking models regarding cellular interactions, and we discuss intrinsic mechanical and biochemical cues that influence cell attachment and phenotype in proliferation and homeostasis. As for future perspectives, micro-physiological models integrating multidisciplinary techniques will be of great potential to replicate complex functions of the native ECM and will require close collaborations between cell biologists, engineers, and materials scientists to push the frontiers of possibility. Fabricating *in vitro* models that can closely mimic the *in vivo* ECM microenvironment features will be cost-effective and sustain batch-to-batch consistency with improved control over different factors. Improving the properties and complexity of ECM mimics would boost future discoveries ranging from fundamental pathology understanding to clinical treatments and preventions.

## Author contributions

A.A. and D.Z. did most writing for the manuscript and literature studying. C.M, M. Y, W. Z discussed and edited the manuscript; H.Y provided the resources; L.M. and Y.Y.S.H. supervised the manuscript process, review and edited the manuscript.

## Ethics approval and consent to participate

Not applicable.

## Declaration of competing interest

Yan Yan Shery Huang is an editorial board member for Bioactive Materials and was not involved in the editorial review or the decision to publish this article. All authors declare that there are no competing interests.
